# fMRI Acoustic Noise Enhances Parasympathetic Activity in Humans

**DOI:** 10.3390/brainsci11111416

**Published:** 2021-10-27

**Authors:** Anna-Lisa Schuler, Giovanni Pellegrino

**Affiliations:** Istituto di Ricovero e Cura a Carattere Scientifico San Camillo Hospital, Via Alberoni 70, 30126 Venice, Italy; giovanni.pellegrino@hsancamillo.it

**Keywords:** HRV, fMRI, auditory processing

## Abstract

Background: Functional magnetic resonance imaging (fMRI) is one of the most important neuroimaging techniques; nevertheless, the acoustic noise of the MR scanner is unavoidably linked to the process of data acquisition. We hypothesized that the auditory noise of the scanner has an effect on autonomic activity. Methods: We measured heart rate variability (HRV) while exposing 30 healthy subjects to fMRI noise. In doing so, we demonstrated an increase in parasympathetic nervous system (PNS) activity compared to silence and white noise and a decrease in sympathetic nervous system (SNS) activity compared to white noise. Conclusions: The influence of MR scanner noise on the autonomic nervous system should be taken into account when performing fMRI experiments.

## 1. Introduction

Resting-state functional magnetic resonance imaging (fMRI) is an invaluable tool for clinical and basic neuroscience [1]. The process of fMRI sequence acquisition is unavoidably linked to high intensity (mostly >80 dB) and rhythmic acoustic noise, typically lasting 5–10 min. Previous studies suggest that fMRI noise may affect brain activity and connectivity, but the potential impact on the autonomic system is poorly known. Interestingly, music and auditory stimuli influence autonomic activity and brain connectivity, as revealed by heart rate variability (HRV) [2,3]. The introduction of noise to the brain has long been shown to have an influence on signal processing, the effects of which have been termed stochastic and coherence resonance [4,5]. We designed an experiment where an electrocardiogram (EKG) was acquired under three conditions: (a) while listening to fMRI acoustic noise (hereafter fMRI), (b) during acoustic White noise exposure (hereafter White) and (c) at rest during silence (hereafter Silence). Our goal was to assess the influence of fMRI acoustic noise on HRV in a within-subject design.

## 2. Materials and Methods

Thirty healthy participants aged 20–50 were included (mean age = 28.57 ± 4.18 years; 24 female). Exclusion criteria were: (a) present or past history of neuropsychiatric disorders, (b) hearing deficits, (c) irregular wakefulness-sleep cycle, (d) medications that act on the central nervous system, and (e) medical conditions potentially affecting the autonomic activity. The study was approved by the Research Ethics Board of the Province of Venice, Italy, complied with the 1964 Declaration of Helsinki and its later amendments and was performed at IRCCS San Camillo Hospital in Venice, Italy. Subjects signed a written informed consent prior to participation.

**Figure 1:**
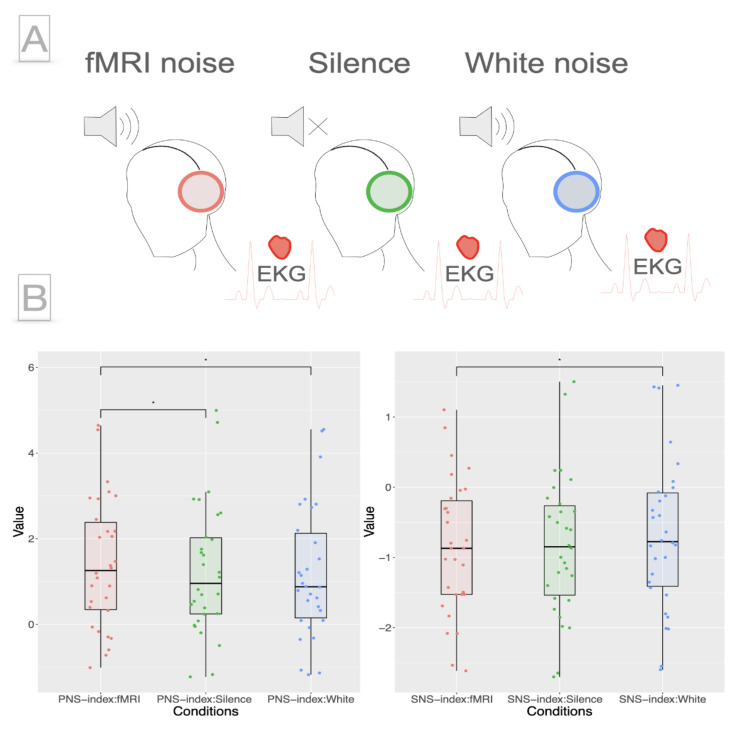
Experimental design and autonomic difference between Silence, fMRI and White noise. Panel **A**. Subjects were exposed to fMRI noise, Silence or White noise in a counterbalanced order during the course of one EKG recording session. Panel **B**. Parasympathetic activity was significantly higher during fMRI noise as compared to Silence and White noise. Sympathetic activity was significantly higher for White noise as compared to fMRI noise.

Participants were lying supine with eyes closed and wearing earplugs connected to the audio delivery system. They were instructed to relax during the experiment. An EKG was recorded with one bipolar electrode on the left and right upper part of the musculus pectoralis. The acoustic noise of the fMRI corresponded to a standard echo planar imaging (EPI) sequence with the following parameters: [TR] = 2000 ms, [TE] = 35 ms, voxel size = 3.3 mm^3^ isotropic, slices = 37, frequency = 0.5 Hz + 54 Hz + ~11 kHz (220 voxel in gradient direction x slices acquisition frequency = 220 × 54 Hz) of a 3T Ingenia CX Philips scanner. The sound pressure level was set to 85 dB during White and fMRI. A depiction of the fMRI noise spectrum can be found in Figure S1. The order of the conditions was counterbalanced across subjects. Each condition lasted 8 min (Figure 1, Panel A).

Kubios software [6] was used to extract metrics for the autonomic nervous system activity from EKG R-R intervals. We specifically focused on two comprehensive measures for sympathetic nervous system (SNS) and parasympathetic nervous system (PNS) activity [7], as defined in the Kubios software (v. 3.5, https://www.kubios.com/) (accessed on 25 October 2021). While the SNS index assumes an increase in heartrate and a decrease in heart rate variability, the PNS index takes into account the opposite pattern [8]. The levels of SNS and PNS were evaluated between the three conditions using repeated measures ANOVA.

## 3. Results

Kolmogorov-Smirnov tests for each condition indicated normal distribution for PNS and SNS variables. Mauchly’s tests indicated the sphericity of data.

Repeated measures ANOVA was significant for PNS (F_2,58_ = 4.129, *p* = 0.021) as well as SNS (F_2,58_ = 4.612, *p* = 0.014).

Consequently, post hoc pairwise paired t-tests were calculated in order to reveal significant differences between conditions.

PNS was significantly higher for fMRI compared to Silence (t_29_ = 2.33, p_fdr_ = 0.04, mSilence = 1.25, mfMRI = 1.41) as well as compared to White (t_29_ = 2.54, p_fdr_ = 0.04, mWhite = 1.19, mfMRI = 1.41; Figure 1, Panel B, left). There was no significant difference in PNS between Silence and White (t_29_ = 0.78, p_fdr_ = 0.44). SNS was significantly higher for White than for fMRI (t_29_ = 2.62, p_fdr_ = 0.04, mWhite = −0.68, mfMRI = −0.85; Figure 1, Panel B, right); there were no differences in the comparisons of fMRI–Silence (t_29_ = 0.63 p_fdr_ = 0.54) and White–Silence (t_29_ = 2.18, p_fdr_ = 0.057) for SNS.

## 4. Discussion

The significant enhancement of PNS and the decrease of SNS during fMRI might reflect the tendency to transition into a relaxed state and drowsiness favored by exposure to the monotonous rhythmic sound environment [9]. People in the MR scanner face difficulties in maintaining a state of wakefulness over the time of the scanning session, with sleep states contaminating resting-state connectivity patterns and 30% of the subjects unable to sustain wakefulness after 3 min [10]. On the other hand, we may speculate that the increase of PNS/decrease in SNS score might be independent of drowsiness and possibly due to the specific noise pattern produced during fMRI acquisition. This interpretation would be in agreement with previous studies, demonstrating the association between autonomic function and auditory processing [2,11]. In this respect, exposure to rhythmic noise might influence the autonomic nervous system via entrainment [5], compared to white noise that might influence the excitability of the brain [12,13].

The effect of fMRI acoustic noise on autonomic activity should therefore not be neglected. The acquisition of an EKG during fMRI should be considered a potential tool to unveil and account for such an effect.

## Data Availability

The data will be made available on reasonable request to the authors.

## Supplementary Materials

The following are available online at https://www.mdpi.com/article/10.3390/brainsci11111416/s1, Figure S1: Signal Spectrum of the fMRI Acoustic Noise Stimulus.
