# Supplementary figures and images for: fMRI Acoustic Noise Enhances Parasympathetic Activity in Humans
Source: Brain Sci. 2021 Oct 27;11(11):1416. doi: 10.3390/brainsci11111416 (PMC8615429; doi:10.3390/brainsci11111416)

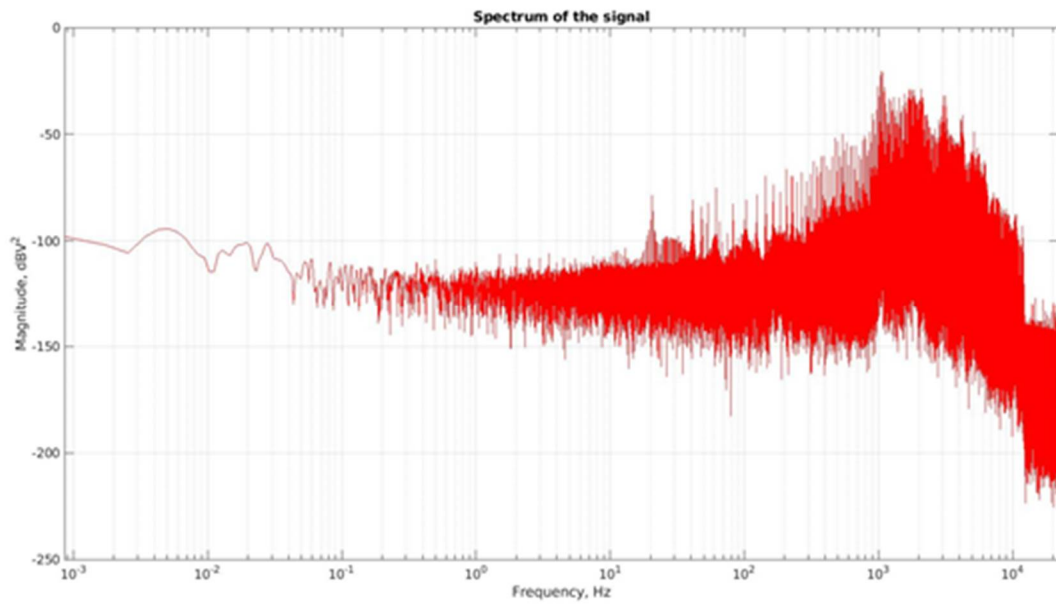

Figure S1. *Signal Spectrum of the fMRI Acoustic Noise Stimulus.*

Supplement: Supplementary file 1 [file brainsci-11-01416-s001.zip › brainsci-1408969-supplementary.pdf]
